# Change in empathic disequilibrium across childhood and associations with socioemotional difficulties

**DOI:** 10.1007/s00787-025-02760-3

**Published:** 2025-06-11

**Authors:** I. Shalev, R. Waller, N. J. Wagner, F. Uzefovsky

**Affiliations:** 1 Psychology Department, Ben-Gurion University of the Negev, 1 David Ben-Gurion Blvd, Be’er Sheva, Israel; 2 Medical Research Council Cognition and Brain Sciences Unit, University of Cambridge, Cambridge, UK; 3 Department of Psychology, University of Pennsylvania, Philadelphia, PA USA; 4 Department of Psychological & Brain Sciences, Boston University, Boston, MA USA

**Keywords:** Empathic disequilibrium, Empathy, Socioemotional outcomes, Child development

## Abstract

Empathy, a socioemotional capacity with emotional and cognitive components, develops from infancy onward. An imbalance between these components, termed empathic disequilibrium, is related to socioemotional difficulties in adulthood, but how empathic disequilibrium changes across development and whether these changes relate to socioemotional difficulties, remain to be discovered. This study investigated changes in empathic disequilibrium with age in children aged 3 to 12 years and its associations with socioemotional outcomes. 303 children from the general population were assessed at three timepoints, four months apart. Parent-reported measures of cognitive and emotional empathy, along with socioemotional outcomes (conduct and emotional problems, callous-unemotional traits, and prosocial behavior), were analyzed. Considering within and between participant variability in age, we characterized empathic disequilibrium development, and examined whether socioemotional outcomes at the last timepoint moderated this trajectory. Empathic disequilibrium typically shifted from emotional-empathy dominance at age 3 years to equilibrium between ages 4.7 and 6.9. Afterwards, a trend toward cognitive-empathy dominance emerged, which declined during early adolescence. Children with socioemotional difficulties at the final timepoint showed distinct developmental patterns of empathic disequilibrium. Specifically, children with high emotional problems, callous-unemotional traits, and low prosocial behavior were less likely to achieve empathic equilibrium by age 12, with those showing high conduct problems reaching equilibrium later than their peers. This study illustrates how empathic disequilibrium develops from early childhood to adolescence and its links with socioemotional outcomes. These findings suggest that tracking empathic disequilibrium could help identify children who may benefit from tailored interventions.

**Supplementary Information:**

The online version contains supplementary material available at 10.1007/s00787-025-02760-3.

Empathy is an early and fundamental socioemotional capacity often linked with positive social and psychological outcomes [1, 2]. Traditionally, empathy is studied as comprising distinct emotional and cognitive components. However, research in adults has emphasized the balance or imbalance between emotional and cognitive components, termed empathic disequilibrium [3]. In adults, empathic disequilibrium has been linked to clinical conditions, sometimes explaining symptom variability better than empathy alone [3–5]. As emotional and cognitive empathy emerge early in life [6], studies need to explore changes in empathic disequilibrium across childhood, which could provide novel insight into the emergence of behavioral and emotional difficulties as well as prosocial behavior [1, 6, 7].

From birth, we are motivated to form social connections, which are underpinned by empathy – the ability to understand and share others’ feelings [1, 8]. Atypical empathy development is linked to socioemotional difficulties [1, 9], including callous-unemotional traits [10], disruptive behavior disorders [7], and poor social competence [11]. Conversely, empathy contributes to adaptive prosocial behavior and moral development [1].

Empathy is a multidimensional construct comprising cognitive and emotional components [2]. Cognitive empathy is the ability to comprehend others’ feelings, whereas emotional empathy is the ability to resonate with others’ feelings while maintaining self-other distinction. These components follow distinct developmental pathways. Emotional empathy emerges by three months [12], increases gradually during the second year of life [13], and remains relatively stable across life [14]. In contrast, cognitive empathy develops later and continues to develop into early adulthood. Unlike emotional empathy, it can be more susceptible to environmental factors, like family or peer interactions [15].

Although studies support the distinction between emotional and cognitive empathy, there are mixed findings regarding their relationships with socioemotional outcomes, including attachment [16], prosocial behavior [17], and aggression [18]. These mixed findings may theoretically reflect that real-life empathic responding requires both cognitive and emotional components to work in tandem [19, 20], a factor often overlooked in empirical studies.

To address this, research has begun to explore the intra-individual interplay between emotional and cognitive empathy, reflected in the balance and imbalance between these components, termed empathic disequilibrium [3]. Existing research on empathic disequilibrium has focused on adults [3, 5, 21], emphasizing two types of disequilibrium, each with unique predictive associations to mental health outcomes, beyond overall level of empathy.

Emotional empathy dominance (i.e., emotional empathy outweighs cognitive empathy) is related to heightened emotional reactivity, thus potentially experienced as overwhelming [5]. Emotional empathy dominance has been linked to alexithymia [3], anxiety symptoms [5], and autism and schizophrenia diagnoses [21, 22]. The second form, cognitive empathy dominance (i.e., cognitive empathy outweighs emotional empathy), is hypothesized to reflect emotional responsiveness that might be perceived as lacking emotional tone or sensitivity [5], and has been associated with depressive symptoms [5], psychopathic traits, and positive symptoms of schizophrenia [22]. Importantly, in some cases, empathic disequilibrium relates more strongly to symptom variability than empathy alone [3, 5, 22].

A major gap remains in understanding how empathic disequilibrium develops across childhood and adolescence. A prior study assessed 13–18 year-olds across three timepoints and found that emotional empathy dominance was related to symptoms of social anxiety [23]. Importantly, changes in empathic disequilibrium over time were independent of changes in overall empathy levels. Since empathy emerges earlier in childhood, including a lag in the development of cognitive relative to emotional empathy [6, 8], studies are needed that investigate developmental trajectories of empathic disequilibrium in younger children. This could provide insight into the emergence of behavioral and emotional difficulties, as well as prosocial behavior.

Thus, the current study aimed to examine differences in empathic disequilibrium across age in children aged 3 to 12 years and explore links between empathic disequilibrium and socioemotional outcomes. Importantly, empathic disequilibrium is normally distributed within community samples of adults, with most individuals showing relatively balanced empathy [5]. However, since emotional empathy is already evident at age 3 years and cognitive empathy develops gradually [6, 8], we hypothesized that the younger children in our sample would exhibit emotional empathy dominance, which would decrease with age. Since empathic disequilibrium has been linked to psychopathology in adults [5], we hypothesized that children with higher emotional problems, behavioral problems, and callous-unemotional traits would show developmental pathways characterized by either greater emotional empathy dominance or cognitive empathy dominance. Finally, since empathy evolved to promote prosocial behavior [1] and as adaptive empathic reaction relies on the mutual functioning of both cognitive and emotional empathy [19, 20], we hypothesized that children with lower prosocial skills would show greater empathic disequilibrium and/or achieve this balance at later ages. As empathic disequilibrium was shown to be sex-sensitive [21], we tested whether sex moderated the developmental trajectory of empathic disequilibrium or associations with socioemotional outcomes.

## Methods

### Participants and procedure

Participants were 303 children (51.83% girls) and their parents (98.02% mothers) who took part in a longitudinal study with three assessment points, each four months apart. All measures described below were assessed at every time point (see Table 1 for demographics, see [24] for more details). Children’s age ranged from 3.01 (at the first timepoint) to 11.59 (at the last timepoint) years (*M*_age_ = 6.43 ± 2.13). The age distribution is presented in Figure S1A. Figure S1B shows the age range for both between- and within-participant variability across the timepoints, indicating complete coverage of the age range. Children were recruited through social media and community flyers in two cities in the United States (Boston and Philadelphia). Children had no history of severe medical disorders or developmental conditions. Parents provided informed consent and completed online surveys via Qualtrics about themselves and their child (reporting on the youngest child if they had multiple children). All procedures were approved by the institutional review boards of Boston University and the University of Pennsylvania.

**Table 1:** Demographic characteristics

	Variable	Statistic
Child	Sex	51.83% girls
	Age at T1	Mean = 6.43 (2.13) years
	Race	
	White	65.68%
	Mixed race	15.51%
	Black or African American	11.88%
	Asian	4.95%
	Other	1.98%
Parent	Age at T1	Mean = 38 (5.21)
	Sex	98.01% mothers
	Race	
	White	73.93%
	Black or African American	10.56%
	Asian	8.25%
	Mixed race	4.95%
	Other	2.31%
	Relationship to the child	
	Biological parent	94.39%
	Foster parent	0.33%
	Adoptive parent	4.62%
	Other	0.66%
	Education	
	Less than High School diploma	0.33%
	High-School diploma/GED	3.97%
	Some college (no degree)	5.96%
	Associate's or vocational/technical degree	4.64%
	Bachelor's degree	24.17%
	Graduate degree	59.94%
	Other	0.99%

### Power analysis

As the sample was used in a previous study [24], an a priori power analysis was not applicable. Since post hoc power analysis based on observed power is highly criticized [25], we instead conducted a Monte Carlo simulation with 5,000 resamples using the existing data structure (e.g., sample size, variance) to estimate the minimum effect size with sufficient power (1-β = 0.80, *α* = 0.05) that is required to detect the most power-demanding effect (interaction effect with the child’s characteristics as a moderator, see below). This analysis, implemented using the simr package v1.0.7 [26], indicated that the study had sufficient power to detect a small effect size (*r* = 0.15).

### Missing data

Overall, 10.09% missing data in key variables was observed with attrition at the third timepoint of 49 participants (16.17%). Table S1 describes missing data in key variables and attrition across the time points. Little’s test for a matrix containing the key variables (that were used for completing missing data) was insignificant (*χ*^2^(61) = 75.06, *p* = 0.11), suggesting missing data is potentially missing completely at random. The missing data was completed using Bayesian multiple imputation via the JointAI package v1.0.5 [27], with *m* = 10 imputations and 5,000 iterations. MCMC chains adequately converged across all models as assessed by visually inspecting the traceplot and a Gelman-Rubin criterion of 1.00.

### Measures

#### Overall empathy

Griffith Empathy Measure [GEM; 14], 23-item parent-reported on a 9-point scale (−4 = “strongly disagree”; 4 = “strongly agree”) was used to measure empathy. The GEM includes 9-item emotional (e.g., “*child becomes sad when other children are sad*”) and 6-item cognitive (e.g., “*child can’t understand why other people get upset*”) empathy subscales. We computed mean scores for each subscale and total scores across all items. Cronbach’s α for emotional and cognitive empathy were 0.83 and 0.70, respectively.

#### Empathic disequilibrium

In this study, empathic disequilibrium is treated as the predicted variable and not as a predictor. While previous studies recommended polynomial regression with response surface analysis to capture empathic disequilibrium [4, 5, 21, 28], there is currently no clear application of this method to predicted variables [29]. Therefore, following previous studies [3, 23], empathic disequilibrium was calculated using difference scores. The means of cognitive empathy and emotional empathy were centered across the entire study period to ensure the two scores were comparable. The standard errors of emotional and cognitive empathy were approximately equal at all time points (Table 2). Empathic disequilibrium was calculated by subtracting cognitive empathy from emotional empathy. Thus, a score of zero would suggest that both cognitive and emotional empathy are similar in level (regardless of whether they are low, high, or average). Negative and positive scores represent emotional empathy dominance and cognitive empathy dominance, respectively.

**Table 2:** Descriptive statistics

	Variable	Timepoint mean (SE)		
		T1	T2	T3
GEM	Overall empathy	5.30 (0.05)	5.35 (0.05)	5.37 (0.05)
	Emotional empathy	4.54 (0.07)	4.67 (0.07)	4.69 (0.07)
	Cognitive empathy	6.04 (0.06)	5.99 (0.07)	6.06 (0.07)
SDQ	Conduct problems	1.50 (0.09)	1.72 (0.10)	1.69 (0.10)
	Emotional problems	2.08 (0.12)	2.10 (0.12)	2.11 (0.13)
	Prosocial behavior	7.81 (0.11)	7.69 (0.11)	7.76 (0.12)
ICU	Total score	17.07 (7.73)	17.06 (8.40)	17.39 (8.37)

Mean raw scores of emotional and cognitive empathy are depicted (before centering the scores). Standard error is depicted in parenthesis. *GEM* Griffith Empathy Measure; *SDQ* Strengths and Difficulties Questionnaire; *ICU* Inventory of Callous-Unemotional Traits

#### Socioemotional difficulties and prosocial behavior

The Strengths and Difficulties Questionnaire [SDQ; 30], a 25-item parent-report questionnaire with a 3-point scale (0 = “not true” to 2 = “certainly true”), was used. We focused on conduct problems (e.g., “*child often fights with other children or bullies them*”), emotional problems (e.g., *child has many fears, easily scared*”), and prosocial behavior (e.g., “*child shared readily with other children*”) subscales, that have established cut-off scores (≥ 4 for conduct problems; ≥ 5 for emotional problems, and ≤ 4 for prosocial behavior). Cronbach’s α for conduct problems, emotional problems, and prosocial behavior were 0.64, 0.72, and 0.72, respectively.

#### Callous-unemotional traits

We assessed child callous-unemotional using the Inventory of Callous-Unemotional Traits [ICU; 31], a 24-item parent-report measure (e.g., “*child seems very cold and uncaring to others*”) scored on a 4-point Likert scale (0 = Not true at all to 3 = Definitely true). We derived total scores by summing all 24 items. The ICU demonstrated good reliability (Cronbach’s α = 0.86).

### Statistical analysis

All analyses were conducted using R v4.3.0 [32]. First, we tested whether age was related to empathic disequilibrium, controlling for overall empathy. Since data were collected from different cohorts during 2020, the first year of the COVID-19 pandemic [33], we controlled for the specific cohort measured to address potential effects stemming from this variability. To address the nested design, we used a 2-level multilevel-modeling (MLM), with age of the child in each of the three time points (level 1) nested within participants (level 2). Within this model, we included a random intercept for participants to account for individual differences in initial empathic disequilibrium. Additionally, we included a random slope for age (and age^2^ when it improved the model fit) to allow the developmental trajectory to change among children. This method allowed us to investigate level-2 age-related trends across the entire age range of our sample, which was the primary focus of our study, while altogether leveraging the short-term within-participant variability of the longitudinal design [similar to 34, 35]. We then tested whether age^2^ improved the model fit and whether child sex and its interaction with age (both linear and curvilinear) contributed to the model. We compared the models using the D1 method, a multivariate Wald test that pools between and within covariance matrices across imputed datasets. Once we identified the best-fitting model, two additional analyses were conducted separately to explore how age also related to emotional empathy, cognitive empathy, and overall empathy. This allowed us to compare these trajectories with that of empathic disequilibrium. Based on the fitted regression lines of the models and their residuals, we estimated the mean age at which empathic disequilibrium balances out for the first time (i.e., emotional and cognitive empathy have the same relative level), as well as its 95% CI, using the *predict* function in R [32].

Second, we examined whether socioemotional difficulties moderated the relationship between empathic disequilibrium and age, using the child’s outcome at the final timepoint as a moderator for age in the best-fitting model, with age and outcome centered. Simple slopes of significant interactions were analyzed using *multcomp* v1.4–25 [36] based on the mean, ± 1 standard deviation from the mean, and the cut-off of the relevant outcome (except for ICU, which does not have a clinical cut-off). If a main effect or interaction effect was found for the characteristic examined, we estimated the mean age at which equilibrium was reached for the first time for each of these groups. The same analyses were repeated for overall empathy, cognitive empathy, and emotional empathy separately (see Supplementary Results).

Additional analyses were conducted to make sure the observed effects are not confounded by differences in age distribution within the sample. Children were categorized into younger and older groups based on the median age. The original model was compared to an expanded model that included interaction terms between age group and all predictors, using multivariate Wald tests.

## Results

Descriptive statistics are in Table 2 and correlations are in Table 3. To estimate the between- and within-subject variability, we calculated intra-class correlation (ICC) for each empathy component. The ICC of empathic disequilibrium was 0.62, indicating both between and within-subject variability. The ICC of overall empathy was 0.74, and cognitive and emotional empathy had ICCs of 0.68 and 0.64, respectively.

**Table 3:** Correlation table

		GEM				SDQ		
		Overall empathy	Cognitive empathy	Emotional empathy	Empathic disequilibrium	Conduct problems	Emotional problems	Prosocial behavior
GEM	Overall empathy	-	-	-	-	-	-	-
	Cognitive empathy	0.66***	-	-	-	-	-	-
	Emotional empathy	0.77***	0.15*	-	-	-	-	-
	Empathic disequilibrium	−0.06	0.67***	−0.63***	-	-	-	-
SDQ	Conduct problems	−0.17**	−0.22***	−0.06	−0.12*	-	-	-
	Emotional problems	0.06	−0.07	0.16*	−0.17**	0.38***	-	-
	Prosocial behavior	0.54***	0.53***	0.22***	0.26***	−0.38***	−0.25***	-
ICU	Callous-unemotional traits	−0.50***	−0.57***	−0.20**	−0.30***	0.53***	0.24***	−0.65***

Zero-order cross-sectional correlation matrix of the third timepoint (the timepoint that the SDQ and ICU scores were used) aiming to provide a snapshot of the cross-sectional correlation. *GEM* Griffith Empathy Measure; *SDQ* Strengths and Difficulties Questionnaire; *ICU* Inventory of Callous-Unemotional Traits. * *p* < 0.05 ** *p* < 0.01, *** *p* < 0.001

### The development of empathic disequilibrium

**Fig. 1:**
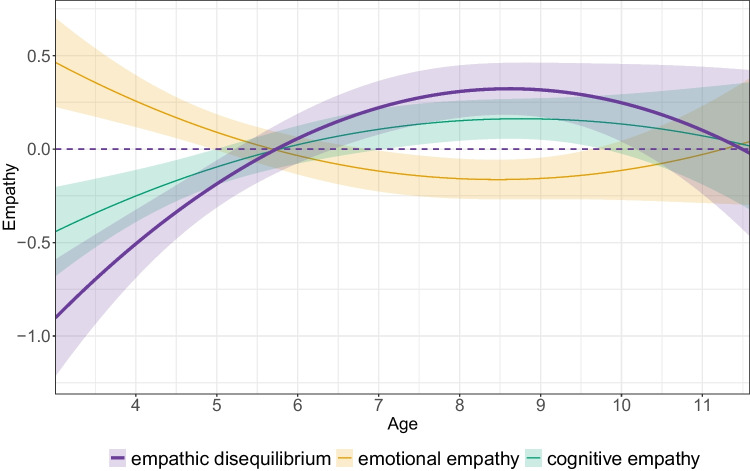
Change in empathic disequilibrium (purple line), emotional (orange line), and cognitive (green line) empathy over age across the entire sample

Residuals of all models were normally distributed. In the youngest ages in the sample (3 years), children exhibited emotional empathy dominance, but this declined significantly over time, as indicated by a linear association with age (*b* = 0.12, 95% CI [0.03, 0.19], *β* = 0.19, *p* < 0.001). That is, with increased age, children approached balance between emotional and cognitive empathy. However, a significant curvilinear association showed that the rate of decline attenuated over time (*b* = −0.03, 95% CI [−0.06, −0.004], *β* = −0.10, *p* = 0.02). The inclusion of the curvilinear association with age significantly improved the model fit (*F* = 4.19, *p* = 0.04) and was incorporated in all models (Table 4, Fig. 1). The mean age for reaching empathic equilibrium was 5.63 [4.70, 6.91]. Separately, both overall empathy and cognitive empathy linearly (but not non-linearly) increased over time (*b* = 0.04, 95% CI [0.01, 0.05], *β* = 0.10, *p* = 0.01 for overall empathy; *b* = 0.07, 95% CI [0.03, 0.12], *β* = 0.15, *p* = 0.002 for cognitive empathy), though emotional empathy was stable over time (Table S2). Sex and its interaction with age were not included in the analyses as they did not improve model fit (*F* = 0.38, *p* = 0.77), indicating no developmental differences between boys and girls. Overall empathy scores were related to empathic disequilibrium (Figure S2).

**Table 4:** Prediction of empathic disequilibrium

	*b*	95% CI	*β*	*p*
Age	**0.12**	**0.03, 0.19**	**0.19**	** < 0.001**
Age^2^	**−0.03**	**−0.06, −0.004**	**−0.10**	**0.02**
Overall empathy	**−0.38**	**−0.52, −0.24**	**−0.21**	** < 0.001**
Cohort	0.13	−0.02, 0.27	0.09	0.08
σ^2^_within-individual variance_	1.44			
σ^2^_Age_	0.07			
σ^2^_Age_^2^	0.04			
σ^2^_r_	0.87

### Variability in empathic disequilibrium and child socioemotional outcomes

**Fig. 2:**
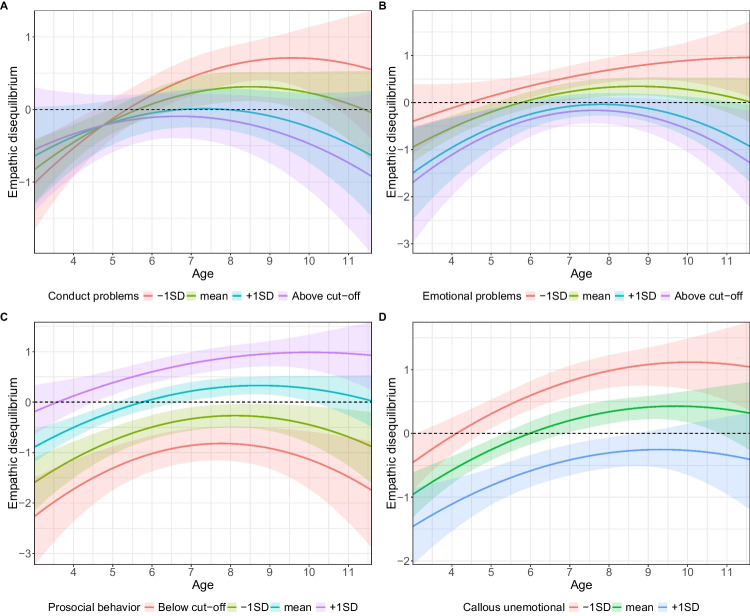
Change in empathic disequilibrium by age across the entire sample, by children’s levels of **A**. conduct problems; **B**. emotional problems; **C**. prosocial behavior; **D**. callous-unemotional traits

First, conduct problems were related to empathic disequilibrium (*b* = −0.12, 95% CI [−0.22, −0.02], *β* = −0.13, *p* = 0.02; see Table S3). The association between age and empathic disequilibrium also varied based on conduct problems (*b* = −0.06, 95% CI [−0.10, −0.02], *β* = −0.13, *p* = 0.01). Specifically, the association between age and empathic disequilibrium was strongest among children with the lowest conduct problems (*b* = 0.22, 95% CI [0.12, 0.31], *β* = 0.31, *p* < 0.001), followed by children with moderate levels (*b* = 0.13, 95% CI [0.06, 0.19], *β* = 0.19, *p* < 0.001). No linear association with age was found for children with high conduct problems (*b* = 0.04, 95% CI [−0.07, 0.14], *β* = 0.06, *p* = 0.46) or those crossing above clinical cut-offs (*b* = −0.003, 95% CI [−0.14, 0.13], *β* = −0.01, *p* = 0.96) (Fig. 2A and Figure S3A). Emotional empathy levels were equal to those for cognitive empathy at a mean age of 5.41 [4.42, 6.27] and 5.67 [4.77, 6.83] for children with low and moderate conduct problems scores, respectively. For children with high conduct problems, the mean age for empathic equilibrium was 7.29, with 95% CI exceeding the age range of this study. The regression line for children above the cut-off did not intersect with the point where emotional and cognitive empathy were equal.

Second, emotional problems were also related to empathic disequilibrium (*b* = −0.13, 95% CI [−0.20, −0.06], *β* = −0.19, *p* < 0.001; see Table S4). However, there was no interaction with age (*b* = −0.02, 95% CI [−0.05, 0.03], *β* = −0.05, *p* = 0.79), suggesting that the decrease in empathic disequilibrium over time did not differ between children with different emotional problems levels. Since the main effect of emotional problems indicates that the intercept varies by children’s characteristics, we examined how empathic disequilibrium changes with age in low, average, high, and above clinical cut-off groups. While children with low and average levels of emotional problems reached equilibrium at the mean age of 4.34 [95% CI: 0, 5.59] and 5.64 [95% CI: 4.71, 6.80], children with high emotional problems and those that crossed the clinical cut-off did not reach, on average, the point where emotional equals cognitive empathy (Fig. 2B). A spaghetti plot is shown in Supplementary Figure S3B.

Third, prosocial behavior at T3 exhibited a strong positive correlation with empathic disequilibrium (*b* = 0.28, 95% CI [0.21, 0.36], *β* = 0.38, *p* < 0.001; see Table S5), but with no significant interaction with age (*b* = 0.01, 95% CI [−0.03, 0.04], *β* = 0.004, *p* = 0.64). Children with moderate levels of prosocial behavior reached equilibrium at an average age of 5.79 [95% CI: 4.98, 6.87], whereas those with high prosocial behavior reached it earlier at 3.34 [95% CI: below the sample range, 4.44]. In contrast, children with lower levels of prosocial behavior or those below the cut-off score did not reach the point of equilibrium. A visual representation is found in Fig. 2C, and a spaghetti plot is presented in Supplementary Figure S3C.

Finally, callous-unemotional traits were also associated with empathic disequilibrium (*b* = −0.07, 95% CI [−0.09, −0.05], *β* = −0.44, *p* < 0.001; see Table S6), though there was no interaction with age (*b* = −0.002, 95% CI [−0.01, 0.01], *β* = −0.03, *p* = 0.55). Children with low callous-unemotional traits reached equilibrium at an average age of 3.84 [95% CI: below sample range, 4.7], and children with average scores did so at 5.98 [95% CI: 5.18, 6.98]. In contrast, children with high callous-unemotional traits did not reach equilibrium (Fig. 2D). A spaghetti plot is displayed in Supplementary Figure S3D.

Including the interaction with age group (younger/older) did not improve model fit for conduct problems (*F* = 0.99, *p* = 0.45), emotional problems (*F* = 0.47, *p* = 0.88), prosocial behavior (*F* = 1.41, *p* = 0.19), or callous-unemotional traits (*F* = 0.94, *p* = 0.48), suggesting that socioemotional strengths and difficulties of children at T3 were consistently related to empathic disequilibrium across both age groups.

## Discussion

We examined the development of empathic disequilibrium across childhood and tested its associations with socioemotional outcomes. As hypothesized, emotional empathy dominance was common at age 3, but decreased with age, with equilibrium observed between ages 4.7 and 6.9 years. After this period, a trend toward cognitive empathy dominance emerged, which declined during early adolescence. Additionally, children's socioemotional difficulties at the final timepoint were associated with the progression of empathic disequilibrium and the time when equilibrium was reached. Children with higher conduct problems, more emotional problems, higher callous-unemotional traits, and lower prosocial behavior generally did not show empathic equilibrium by age 12 (or, in the case of high conduct problems, reached equilibrium later).

Consistent with previous research [8, 13], we showed that cognitive empathy increases with age, while emotional empathy, which typically matures by age 3 (i.e., the youngest age in our sample), remained stable [8, 13, 14]. These patterns do not directly translate to changes in empathic disequilibrium because the latter refers to changes in the balance between the two empathy components, irrespective of their mean-level change. Nevertheless, our findings support that emotional empathy dominance is typical in early childhood, and with time, empathic disequilibrium decreases, with children reaching equilibrium around age 5.61 years. Notably, around this age, abilities like Theory of Mind, self-other distinction, and engagement in pretend play mature [37–39], suggesting that the balance between cognitive and emotional empathy might depend on these earlier developmental milestones.

Unexpectedly, after empathic equilibrium was reached, cognitive empathy continued to increase relative to emotional empathy, and cognitive empathy dominance became typical in older children. This dominance may diminish as children show equilibrium again by early adolescence. Our results suggest that both forms of empathic disequilibrium are typical at different developmental stages. Interestingly, greater variability in cognitive and emotional skills during early childhood, including social interactions and emotional expression, is thought to promote early practice of regulatory strategies, enabling adaptive and flexible use of these strategies later in life [40]. Thus, experiencing both forms of disequilibrium throughout development may help children practice a range of responses, enabling them to adjust their reactions to others’ emotions in different contexts when they grow up. For example, relying on emotion sharing rather than understanding (reflected in emotional empathy dominance), may be adaptive when heightened sensitivity to subtle emotional cues is required [41]. In other cases, a cognitive-focused response, centered on understanding more than feeling, might be beneficial in situations demanding rational problem-solving and practical support [42]. Future studies should examine how early childhood variability may influence flexibility in empathic reactions in adulthood.

Contrary to our hypothesis, empathic disequilibrium was modestly related to overall empathy (Figure S2), with lower levels of overall empathy related to higher cognitive empathy dominance and higher levels related to emotional empathy dominance. Previous studies in adolescence [23] and adulthood [3] found no such relationship. This pattern, where related constructs become independent with age, can point to a shared developmental origin that differentiates over time, similar to the proposed distinction between general and specific intelligence abilities [43]. This suggests that overall empathy and empathic disequilibrium originate from the same developmental or biological origin, becoming more differentiated as these constructs mature.

Relatedly, we found no evidence that empathic disequilibrium differs between boys and girls. Previous studies, however, consistently found that empathic disequilibrium is sex-sensitive later in life [3, 5, 23]. This aligns with evidence that sex differences in many psychological constructs can emerge or become more pronounced during adolescence, likely due to hormonal changes [44].

Examining empathic disequilibrium can provide valuable clinical insights. For example, it may be more informative for understanding socioemotional difficulties in children than levels of emotional and cognitive empathy alone. Our findings suggest that children who demonstrated greater or more persistent periods of emotional empathy dominance later showed more conduct and emotional problems, as well as callous-unemotional traits. The prolonged duration of emotional empathy dominance may contribute to overwhelming experiences due to heightened emotional arousal [5], potentially increasing the children’s risk for developing further behavioral and emotional problems [45].

Tracking empathic disequilibrium throughout development may also shed light on the development of prosocial behavior, as children who exhibited higher prosocial behavior tended to reach equilibrium earlier. It may also emphasize the adaptive role of cognitive empathy dominance, as children with higher prosocial behavior appear to develop this tendency earlier and sustain it for a longer period. Notably, previous research linked cognitive empathy dominance to depressive symptoms [5], so future studies should investigate both the potential benefits and risks associated with this tendency.

### Limitations

Although this study has several strengths, including its longitudinal design and large sample size, some limitations should be addressed in future research. First, all measures relied on parental reports. Combining and comparing these with self-reports could be beneficial as parents and children may capture different aspects of the children’s emotions and behaviors [46]. Moreover, using difference scores limits the interpretation of our findings, since these are often less reliable than their individual components [29]. However, previous studies on empathic disequilibrium reported comparable and replicable results when using both difference scores and more robust and reliable methods that address their limitations [3, 5, 21]. Additionally, the ICC values in this study may suggest similar reliability for empathic disequilibrium, emotional, and cognitive empathy. Nonetheless, behavioral measures of empathy, or the development of tools that directly assess empathic disequilibrium, should enhance the validity of our findings.

Additionally, our investigation was limited to children aged three to twelve. Age three should be of particular interest as this is when emotional empathy matures whereas cognitive empathy still develops [13, 14]. Therefore, before this age, both overall empathy and empathic disequilibrium might be less stable. However, future research should explore empathic disequilibrium across a broader age range and incorporate a longitudinal design with longer follow-up periods. Finally, while our sample was relatively diverse, future studies should also examine the development of empathic disequilibrium in clinical populations.

## Conclusions

The current study suggests that empathic disequilibrium towards emotional empathy dominance is typical in early childhood, with equilibrium typically reached around age six. Moreover, children with emotional and behavioral problems show different patterns of empathic disequilibrium. Recognizing empathic disequilibrium can encourage caregivers and professionals to better understand children's socioemotional difficulties. By doing so, caregivers can better appreciate the strengths and challenges associated with the child’s empathy, including the potentially overwhelming experiences that may accompany it. This may help explain why some children struggle or react with heightened emotional and behavioral problems when responding to others' emotions, while others may be more inclined to offer help.

## Supplementary Information

Below is the link to the electronic supplementary material.

Supplementary file1 (DOCX 15 KB)

Supplementary file2 (DOCX 2310 KB)

## Data Availability

Data in anonymized form and analysis script are publicly available at https://osf.io/hkd8b/?view_only=4bee465f0e36437182f0f3cf4feb9ccc.
