# Supplementary material for: Change in empathic disequilibrium across childhood and associations with socioemotional difficulties
Source: Eur Child Adolesc Psychiatry. 2025 Jun 11;34(11):3655–64. doi: 10.1007/s00787-025-02760-3 (PMC12647174; doi:10.1007/s00787-025-02760-3)
Supplement: Supplementary file 1 — Supplementary file1 (DOCX 15 KB) [file 787_2025_2760_MOESM1_ESM.docx]

**Supplementary Results**

**Variability in empathy and child socioemotional outcomes**

All analyses reported in the main article examining how socioemotional difficulties vary in age (see **Variability in empathic disequilibrium and child socioemotional outcomes** in the Results section) were repeated using overall empathy, emotional empathy, and cognitive empathy.

Conduct problems predicted lower overall empathy (*b* = -.11, 95% CI [-.17, -.05], *β* = -.21, *p* < .001), but the relationship between age and overall empathy did not differ by conduct problems levels (*b* = .01, 95% CI [-.01, .05], *β* = .05, *p* = .25). Examining cognitive and emotional empathy separately, conduct behavior predicted lower levels of cognitive empathy (*b* = -.17, 95% CI [-.25, -.10], *β* = -.24, *p* < .001), but did not predict emotional empathy (*b* = -.07, 95% CI [-.15, .01], *β* = -.10, *p* = .08), and for both cognitive and emotional empathy, conduct problems did not interact with age (*b* = -.02, 95% CI [-.07, .01], *β* = -.07, *p* = .29 for cognitive empathy; *b* = .04, 95% CI [-.001, .08], *β* = .12, *p* = .24 for emotional empathy).

Children’s emotional problems also predicted cognitive empathy (*b* = -.06, 95% CI [-.12, -.003], *β* = -.12, *p* = .04) and emotional empathy (*b* = .08, 95% CI [.03, .14], *β* = .17, *p* = .004), yet it did not predict overall empathy (*b* = .02, 95% CI [-.03, .06], *β* = .05, *p* = .47).

Callous-unemotional traits were related to lower cognitive empathy (*b* = -.07, 95% CI [-.08, -.06], *β* = -.55, *p* < .001), emotional empathy (*b* = -.02, 95% CI [-.04, -.01], *β* = -.17, *p* = .001), and overall empathy (*b* = -.04, 95% CI [-.05, -.04], *β* = -.47, *p* < .001).
