# Supplementary material for: Change in empathic disequilibrium across childhood and associations with socioemotional difficulties
Source: Eur Child Adolesc Psychiatry. 2025 Jun 11;34(11):3655–64. doi: 10.1007/s00787-025-02760-3 (PMC12647174; doi:10.1007/s00787-025-02760-3)
Supplement: Supplementary file 2 — Supplementary file2 (DOCX 2310 KB) [file 787_2025_2760_MOESM2_ESM.docx]

**Supplementary Tables**

**Table S1. Number of participants and missing data in each key variable across time**

|  | **T1** | **T2** | **T3** | **overall observations (%missing)** |
| --- | --- | --- | --- | --- |
| Participants | 303 (0%) | 269 (11.22%) | 254 (16.17%) | 826 (9.13%) |
| GEM | 303 (0%) | 266 (12.21%) | 254 (16.17%) | 823 (9.46%) |
| SDQ | 303 (0%) | 267 (11.88%) | 254 (16.17%) | 824 (9.35%) |
| ICU | 299 (1.32%) | 267 (11.88%) | 254 (16.17%) | 820 (9.79%) |
| Age | 303 (0%) | 269 (11.22%) | 254 (16.17%) | 826 (9.13%) |
| Child’s sex | 301 (.66%) | - | - | 301 (.66%) |

Number of participants and completed key variable within each timepoint (T1-T3). For T1 to T3, the percentage of cumulative attrition is in parenthesis. Overall missing observation (and its percentage) is shown in the rightmost column. GEM – Griffith Empathy Measure; SDQ – Strengths and Difficulties Questionnaire; ICU – Inventory of Callous-Unemotional Traits.

**Table S2. Prediction of cognitive empathy, emotional empathy, and overall empathy**

|  | **Emotional empathy** | | | | **Cognitive empathy** | | | | **Overall empathy** | | | |
| --- | --- | --- | --- | --- | --- | --- | --- | --- | --- | --- | --- | --- |
|  | ***b*** | **95% CI** | ***β*** | ***p*** | ***b*** | **95% CI** | ***β*** | ***p*** | ***b*** | **95% CI** | ***β*** | ***p*** |
| Age | -.03 | -.09, .01 | -.08 | .16 | .07 | **.03, .12** | **.15** | **.002** | **.04** | **.01, .05** | **.10** | **.01** |
| Age^2^ | .02 | -.01, .03 | .04 | .18 | -.02 | -.05, .01 | -.08 | .22 | -.004 | -.02, .01 | -.03 | .51 |
| Cohort | -.09 | -.20, .01 | -.09 | .10 | .04 | -.07, .15 | .04 | .44 | -.03 | -.11, .05 | -.04 | .43 |
| σ^2^_ID_ | .73 |  |  |  | .76 |  |  |  | .46 |  |  |  |
| σ^2^_Age_ | .03 |  |  |  | .06 |  |  |  | .007 |  |  |  |
| σ^2^ _Age_^2^ | .02 |  |  |  | .01 |  |  |  | .003 |  |  |  |
| σ^2^_r_ | .66 |  |  |  | .62 |  |  |  | .39 |  |  |  |

**Table S3. Prediction of empathic disequilibrium developmental path by conduct problems**

|  | ***b*** | **95% CI** | ***β*** | ***p*** |
| --- | --- | --- | --- | --- |
| **Age** | **.14** | **.07, .19** | **.19** | **< .001** |
| **Age^2^** | **-.04** | **-.06, -.01** | **-.11** | **.01** |
| **Age X conduct problems** | **-.06** | **-.10, -.02** | **-.13** | **.01** |
| Age^2^ X conduct problems | .001 | -.01, .01 | .01 | .73 |
| **Overall empathy** | **-.41** | **-.55, -.26** | **-.22** | **< .001** |
| **Conduct problems** | **-.12** | **-.22, -.02** | **-.13** | **.02** |
| Cohort | .07 | -.05, .24 | .06 | .18 |
| σ^2^_ID_ | 1.13 |  |  |  |
| σ^2^_Age_ | .10 |  |  |  |
| σ^2^ _Age_^2^ | .04 |  |  |  |
| σ^2^_r_ | .87 |  |  |  |

**Table S4. Prediction of empathic disequilibrium developmental path by emotional problems**

|  | ***b*** | **95% CI** | ***β*** | ***p*** |
| --- | --- | --- | --- | --- |
| **Age** | **.12** | **.08, .19** | **.21** | **< .001** |
| **Age^2^** | **-.03** | **-.06, -.01** | **-.11** | **.01** |
| Age X emotional problems | -.02 | -.05, .03 | -.05 | .79 |
| Age^2^ X emotional problems | -.01 | -.02, .001 | -.07 | .09 |
| **Overall empathy** | **-.36** | **-.50, -.22** | **-.20** | **< .001** |
| **Emotional problems** | **-.13** | **-.20, -.06** | **-.19** | **< .001** |
| Cohort | .11 | -.02, .25 | .08 | .11 |
| σ^2^_ID_ | 1.14 |  |  |  |
| σ^2^_Age_ | .03 |  |  |  |
| σ^2^ _Age_^2^ | .03 |  |  |  |
| σ^2^_r_ | .88 |  |  |  |

**Table S5. Prediction of empathic disequilibrium developmental path by levels of prosocial behavior**

|  | ***b*** | **95% CI** | ***β*** | ***p*** |
| --- | --- | --- | --- | --- |
| **Age** | **.14** | **.09, .19** | **.20** | **< .001** |
| **Age^2^** | **-.03** | **-.06, -.01** | **-.10** | **.01** |
| Age X prosocial behavior | .01 | -.03, .04 | .004 | .64 |
| Age^2^ X prosocial behavior | .01 | -.003, .02 | .06 | .20 |
| **Overall empathy** | **-.58** | **-.72, -.43** | **-.31** | **< .001** |
| **Prosocial behavior** | **.28** | **.21, .36** | **.38** | **< .001** |
| Cohort | .08 | -.05, .21 | .06 | .20 |
| σ^2^_ID_ | .98 |  |  |  |
| σ^2^_Age_ | .04 |  |  |  |
| σ^2^ _Age_^2^ | .03 |  |  |  |
| σ^2^_r_ | .87 |  |  |  |

**Table S6. Prediction of empathic disequilibrium developmental path by levels of callous unemotional traits**

|  | ***b*** | **95% CI** | ***β*** | ***p*** |
| --- | --- | --- | --- | --- |
| **Age** | **.16** | **.11, .23** | **.25** | **< .001** |
| **Age^2^** | **-.03** | **-.06, -.002** | **-.11** | **.01** |
| Age X callous unemotional | -.002 | -.01, .01 | -.03 | .55 |
| Age^2^ X callous unemotional | -.0005 | -.003, .002 | -.01 | .76 |
| **Overall empathy** | **-.59** | **-.73, -.45** | **-.32** | **< .001** |
| **Callous unemotional** | **-.07** | **-.09, -.05** | **-.44** | **< .001** |
| Cohort | .04 | -.09, .16 | .03 | .59 |
| σ^2^_ID_ | .95 |  |  |  |
| σ^2^_Age_ | .04 |  |  |  |
| σ^2^ _Age_^2^ | .02 |  |  |  |
| σ^2^_r_ | .87 |  |  |  |

**Supplementary Figures**

**Fig S1. Age distribution across the sample**

**
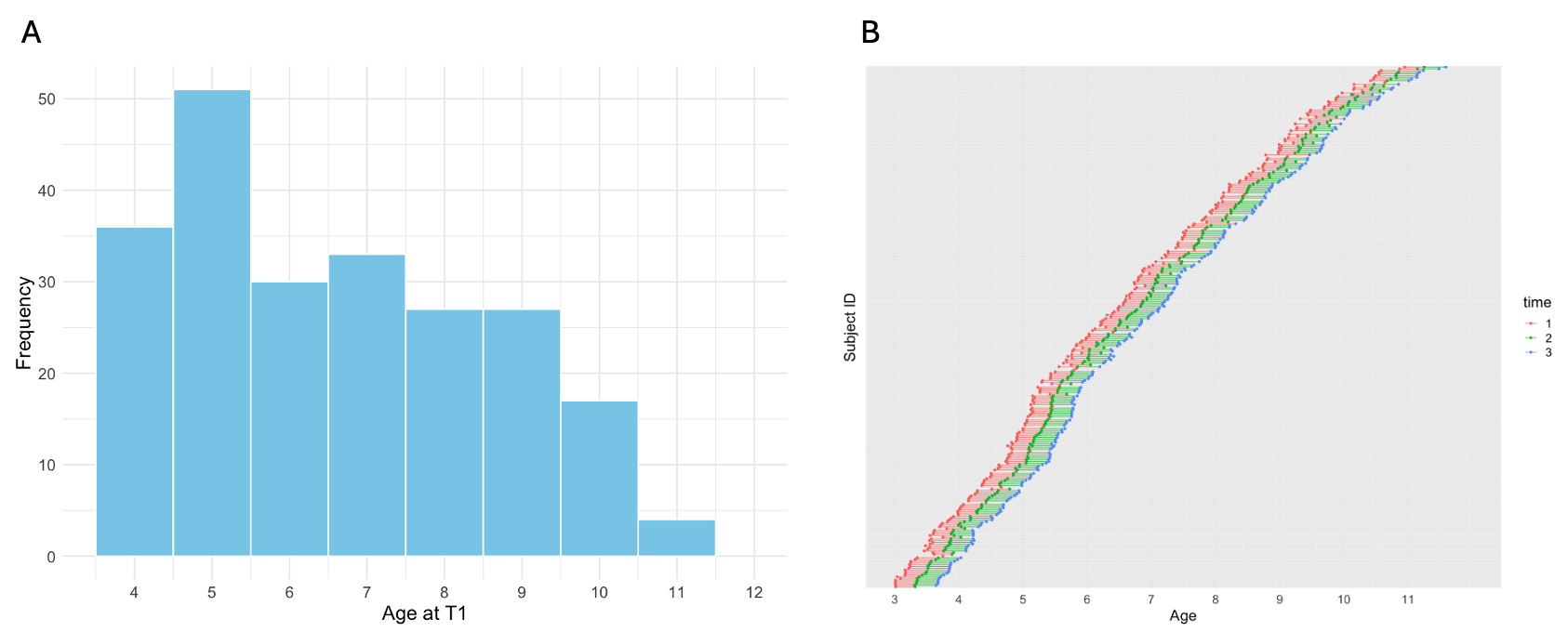
**

**A.** Histogram showing the age distribution at the first time point. **B.** Participants ordered by ascending age, illustrating age coverage across time points. Different colors represent each time point.

**Fig S2. Regression plot of the association between overall empathy and empathic disequilibrium**

**
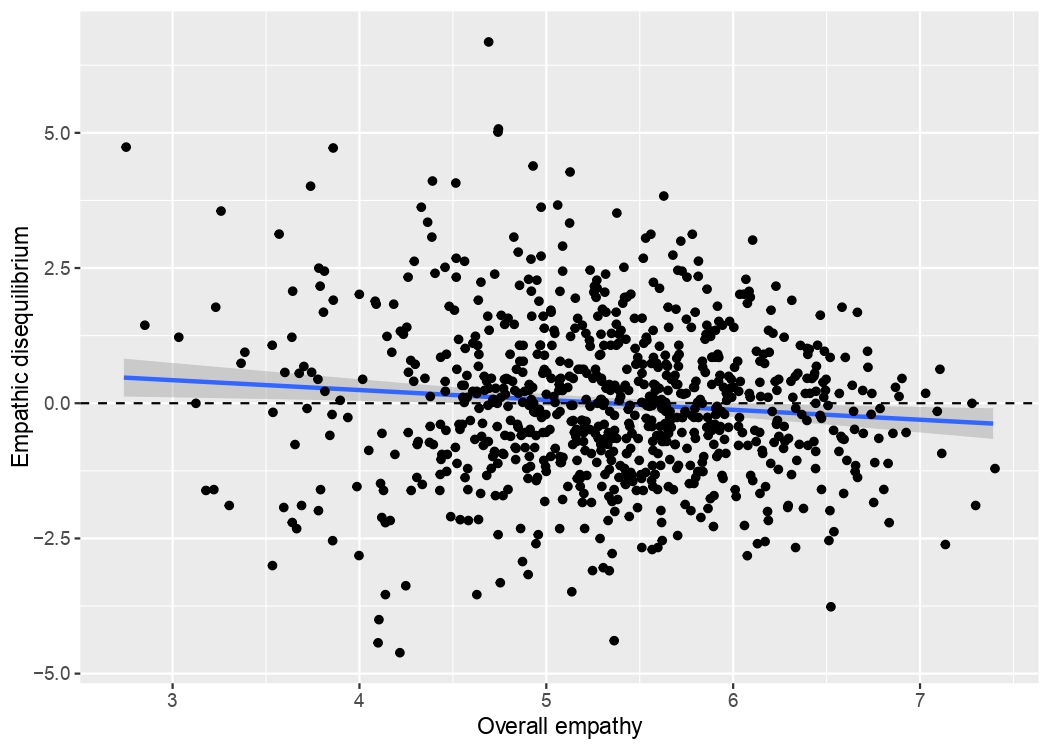
**

**Fig S3. Empathic disequilibrium across ages by children's characteristics**

Spaghetti plots of empathic disequilibrium by age categorized by children’s levels of A. conduct problems; B. emotional problems; C. prosocial behavior; D. callous-unemotional traits.
